# HTRA1 promotes EMT through the HDAC6/Ac‐α‐tubulin pathway in human GBM cells

**DOI:** 10.1111/cns.14605

**Published:** 2024-02-09

**Authors:** Wenbo Zhao, Yibo Wu, Shuai Wang, Feihu Zhao, Wenyu Liu, Zhiyi Xue, Lin Zhang, Jian Wang, Mingzhi Han, Xingang Li, Bin Huang

**Affiliations:** ^1^ Department of Neurosurgery, Cheeloo College of Medicine and Institute of Brain and Brain‐Inspired Science, Qilu Hospital Shandong University Jinan China; ^2^ Jinan Microecological Biomedicine Shandong Laboratory and Shandong Key Laboratory of Brain Function Remodeling Jinan China; ^3^ University of Pittsburgh Medical Center Hillman Cancer Center Pittsburgh Pennsylvania USA; ^4^ Department of Clinical Laboratory Qilu Hospital of Shandong University Jinan China; ^5^ Department of Biomedicine University of Bergen Bergen Norway

**Keywords:** glioma cell migration, glioma cell proliferation, HTRA1, tubulin acetylation

## Abstract

**Background:**

The infiltrative nature of human gliomas renders complete surgical removal of tumors futile. Thus, illuminating mechanisms of their infiltrative properties may improve therapies and outcomes of glioma patients.

**Methods:**

Comprehensive bioinformatic analyses of PRSS family were undertaken. Transfection of HTRA1 siRNAs was used to suppress HTRA1 expression. CCK‐8, EdU, and colony formation assay were employed to assess cell viability, and cell migration/invasion was detected by transwell, wound healing, and 3D tumor spheroid invasion assays. Immunoprecipitation was applied to study the mechanism that HTRA1 affected cell migration. In addition, in situ xenograft tumor model was employed to explore the role of HTRA1 in glioma growth in vivo.

**Results:**

HTRA1 knockdown could lead to suppression of cell viability, migration and invasion, as well as increased apoptosis. Immunoprecipitation results indicates HTRA1 might facilitate combination between HDAC6 and α‐tubulin to enhance cell migration by decreasing α‐tubulin acetylation. Besides, HTRA1 knockdown inhibited the growth of xenografts derived from orthotopic implantation of GBM cells and prolonged the survival time of tumor‐bearing mice.

**Conclusion:**

Our results indicate that HTRA1 promotes the proliferation and migration of GBM cells in vitro and in vivo, and thus may be a potential target for treatment in gliomas.

## Graphical abstract

The HTRA1 serine protease has been found to have the ability to promote glioma cell proliferation, migration/invasion, and to inhibit tumor apoptosis, in vitro and in vivo. HTRA1 might facilitate the combination between HDAC6 and α‐tubulin to reduce α‐tubulin acetylation, enhancing cell migration.

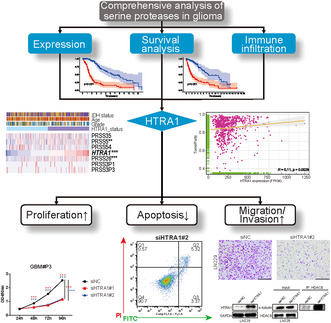

## 1 INTRODUCTION

Human glioma is the most common and lethal intracranial tumors and account for up to 80% of primary human brain tumors. To date, glioma remain one of the most refractory human tumor types due to the scarcity of approaches to manage glioma. One of the most intriguing features of glioma is the frequent intracranial infiltration while extracranial metastasis is rare. In addition, intracranial recurrence after surgery mainly arises from the infiltrative nature of human glioma. Therefore, the study of the mechanisms underlying glioma invasion and infiltration may be beneficial to the development of therapies with improved efficacy in the treatment of this deadly tumor type.

High‐temperature requirement‐A1 serine protease (HTRA1), also named as serine protease 11 (PRSS11), is one of the proteins participating in tumor invasion and migration.^1^ As a member of the serine protease superfamily, HTRA1 is a highly conserved protein expressed in different organisms, including bacteria,^2^ plants, fungi, and human being.^3^, ^4^ HTRA1 is widely expressed in various tissues and organs of human, mostly in the brain, mature epidermis and placenta.^5^ HTRA1 is a 50‐kDa protease with protease and PDZ domains at the C‐terminus.^6^, ^7^ The protein is secreted by cells and involved in extracellular matrix degradation.^8^ It is also reported that HTRA1 could participate intracellularly in cell apoptosis, migration, and invasion in tumor cells through EGFR pathways.^9^, ^10^ Other signaling pathways, including the Wnt,^11^ Notch,^7^ NF‐κB^12^ and TGF‐β^13^, ^14^, ^15^ pathways can also be modulated by HTRA1. On the one hand, HTRA1 is primarily viewed as a tumor suppressor protein in cancer, including ovarian cancer,^16^, ^17^ melanoma,^3^ endometrial cancer^18^, ^19^ and thyroid tumors.^20^ On the other hand, it has also been reported to function as an oncogenic protein facilitating angiogenesis,^7^ tumor proliferation, and metastasis.^21^ In addition, high HTRA1 expression has been associated with poor prognosis in gastric cancer patients.^12^ However, to the best of our knowledge, no other studies to date have illuminated the function of HTRA1 in human glioma.

In this study, we analyzed publicly available databases to explore the expression pattern of HTRA1 in glioma tissues and the correlation between HTRA1 expression levels and the prognosis of glioma patients. We also investigated the function of HTRA1 in glioblastoma (GBM) cells in vitro and in vivo, and found that HTRA1 promoted tumor progression by enhancing proliferation, invasion, and migration. The prometastatic effect of HTRA1 might be mediated by the HDAC6/Ac‐α‐tubulin pathway. In vivo experiments with isogenic HTRA1 knockdown cells indicated that HTRA1 facilitated the infiltration of glioma and shortened the survival period of nude mice. In summary, our findings suggested that HTRA1 functioned as an oncogene in glioma and might be a potential prognostic marker and therapeutic target for gliomas.

## 2 MATERIALS AND METHODS

### 2.1 Clinical specimens and databases

Paraffin‐embedded WHO grade II (*n* = 4), III (*n* = 4), and IV (*n* = 4) glioma tissues were obtained from the Neurosurgery Department of Qilu Hospital (Jinan, China). Normal brain tissues (*n* = 4) were obtained during decompression surgery performed on patients who had sustained severe head trauma. Written informed consent was obtained from patients for research purposes, and all procedures approved by the Ethics Committee of Qilu Hospital. The transcriptome data and patient survival were obtained from TCGA, Rembrandt, and GEPIA2^22^ databases. TCGA and Rembrandt databases were displayed in GlioVis.^23^

### 2.2 Survival analysis and risk score model establishment

The schematic illustration of this process is displayed in Figure S1A. After the 52 members of PRSS family genes were included, survival analysis was used to find PRSS genes whose expression was significantly correlated with prognosis of glioma patients. Then the obtained genes were applied to least absolute shrinkage and selection operator (LASSO) regression to select major PRSS genes contributing to the risk score together with their corresponding coefficients. Then the “predict” function of “glmnet” package in R was employed to calculate the risk score of each sample in TCGA glioma or GBM cohort based on each major genes' expression level and corresponding coefficients.^24^ Basically, it was calculated by the following formula:

$$\begin{array}{c} a=\sum_{k=1}^{n}\left({\text{gene}}_{k}*{\text{coefficient}}_{k}\right),\text{Risk score}=e^{a},\text{where}\;e\;\text{is the natural constant}. \end{array}$$

The patients with a risk score higher than the median number of risk scores were regarded as “high risk”, and the others were considered as “low risk”.

The test and train data were composed of samples randomly selected from all glioma or GBM samples and the test or train data had 50% of all data. Risk scores of each sample were calculated with the major genes' coefficients and their expression level. The prognostic effect of risk models was inspected with survival curve or ROC curve.

### 2.3 Implantation and in vivo imaging of in situ tumors

All animal procedures were approved by the Institutional Animal Care and Use Committee of Shandong University (ID: DWLL‐2021‐101). Cells (1 × 10^6^) expressing luciferase and infected with Lenti‐Control or Lenti‐shHTRA1 were implanted into the cortex of the brains of nude mice. The precise position was 2 mm lateral, 1 mm posterior to the bregma at a depth of 1.5 mm. In vivo imaging was conducted as previously described^25^ with a bioluminescence imaging system (IVIS Lumina Series III; PerkinElmer; MA, USA) at 3, 7, 14, 21, and 28 days after implantation.

### 2.4 Statistical analysis

The Shapiro–Wilk test was used to assess the normality of the data. For comparison of two groups of data, Student's *t*‐test was used for data that passed the normality test, while the Mann–Whitney test was applied to data that did not pass the normality test. The one‐way ANOVA test was applied to the comparison of three or more groups, and Kruskal‐Wallis test was employed when data did not pass normality test. Relationships between variables were analyzed via Pearson's correlation analysis. The results of the CCK‐8 proliferation assay were assessed with two‐way ANOVA, and Dunnett's multiple comparisons test was used for the post hoc test. Survival analysis was conducted by log‐rank test. All values are presented as the means ± SEM. All statistical analyses were performed in GraphPad Prism (version 8.3.0) or R (version 4.1.3).

Detailed Methods are available in Data S1.

## 3 RESULTS

### 3.1 High HTRA1 expression is correlated with increasing grade of glioma

PRSS family members showed highly various functions modulating immunity, carcinogenesis, and cancer progression.^26^, ^27^ The members in this family have been reported to show pro‐tumor effects in prostate cancer,^28^ diffuse large B‐cell lymphoma,^27^ pancreatic cancer,^29^ colorectal cancer^30^ and gastric cancer.^31^ Considering there was no other studies reporting their function in glioma to the best of our knowledge, we tried to study the effect of PRSS family in glioma via bioinformatic tools. All 52 PRSS family genes in TCGA database were included. A risk score was established by applying lasso regression to the PRSS family genes as described in section 2.2 and Figure S1A. The results suggested that patients with high risk scores showed more death and worse prognosis both in all glioma (Figure S1) as well as GBM data (Figure S2). These analyses indicated that PRSS genes' expression might be a prognostic factor in glioma patients. Then we found the expression level of HTRA1 was the highest among 52 serine proteases and expression level of HTRA1 in gliomas was significantly higher than in normal tissues, and high HTRA1 expression predicted poor prognosis of glioma patients (Figure S3). Besides, high HTRA1 expression was found to be associated with low M1 macrophage infiltration (Figure S4A), low immune reactivity and high tumor purity (Figure S4B,C) in TCGA glioma samples. This suggested high HTRA1 expression might be correlated with suppressed anti‐tumor immunity.

**FIGURE 1:**
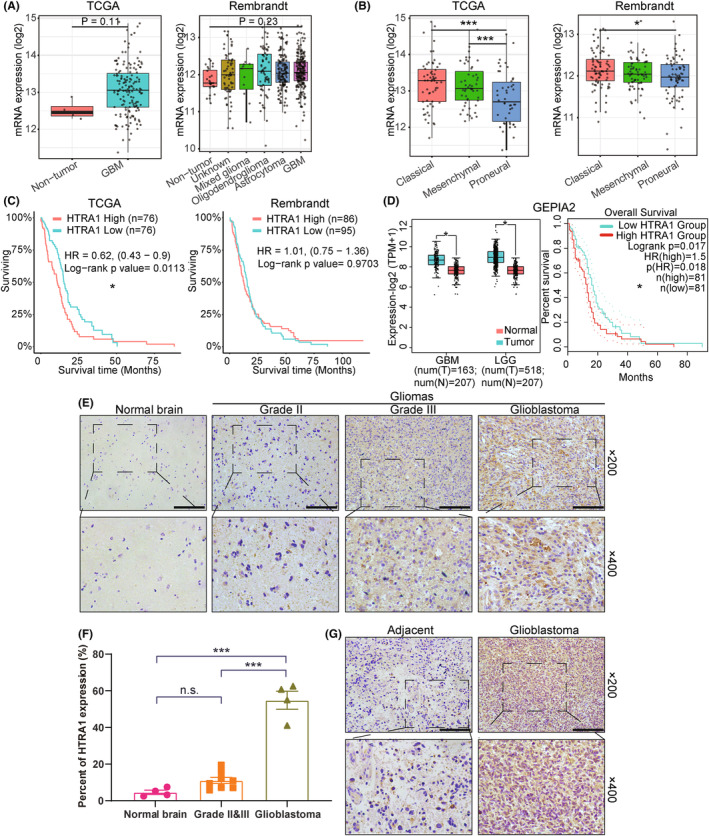
Increased expression of HTRA1 is associated with high grade gliomas and worse prognosis in patients. (A, B) Expression levels of *HTRA1* based on analysis of TCGA (*n* = 667) and Rembrandt (*n* = 537) databases displayed by (A) histology or (B) molecular subtype. (C) Survival rate of GBM patients with high or low *HTRA1* expression levels based on analysis of the TCGA (left, *n* = 152) or Rembrandt (right, *n* = 181) database. (D) Expression levels of HTRA1 (GBM, *n* = 163; LGG, *n* = 518) and survival rate of GBM patients (*n* = 162) with high or low *HTRA1* expression levels in GEPIA2 database. (E, F) Representative images of immunohistochemistry detecting HTRA1 in different grades of glioma and normal brain tissue. Bar graph of the percent of cells expressing HTRA1. Bar = 100 μm. (G) Representative images of immunohistochemistry detecting HTRA1 in glioblastoma and adjacent brain tissues. Bar = 100 μm. (A, B and F), one‐way ANOVA, post hoc Dunnett's test; (C) and survival analysis in (D), log‐rank test; Expression analysis in (D), Student's *t*‐test. *P < 0.05; ***P < 0.001.

To explore the role of HTRA1 in glioma, analysis was performed on publicly available databases to determine the expression levels of HTRA1 in human gliomas. Analysis was first performed in the GlioVis data portal^23^ to determine the expression levels of *HTRA1* and the possible correlation of overall survival with glioma patients (Figure 1A–C). The average *HTRA1* expression level was slightly higher in GBM samples than in nontumor samples in the TCGA (Figure 1A, left) and Rembrandt datasets (Figure 1A, right). *HTRA1* expression levels were also significantly higher in classical and mesenchymal subtypes than in the proneural subtype in the TCGA (Figure 1B, left) and Rembrandt datasets (Figure 1B, right). Both classical and mesenchymal subtypes are regarded as more aggressive than the proneural subtype.^32^ High *HTRA1* expression was also significantly correlated with worse prognosis in glioma patients in the TCGA cohort (Figure 1C). In the Rembrandt cohort, the patients with high *HTRA1* expression also showed a slightly shorter median overall survival than patients in the low expression group (Figure 1C, right). *HTRA1* also showed increased expression in low‐grade glioma (LGG) and GBM compared to normal brain tissue samples in another public database, GEPIA2 (Figure 1D, left). In this dataset, patients with tumors with high *HTRA1* expression exhibited worse prognosis than patients with tumors of low *HTRA1* expression (Figure 1D, right).

Immunohistochemistry furthermore showed that HTRA1 protein levels in GBM were significantly higher than in adjacent normal tissue and thus paralleled the RNA analysis (Figure 1E–G). Overall, we demonstrated that HTRA1 expression levels were significantly increased in higher grade gliomas and that high expression was correlated with worse prognosis in glioma patients.

### 3.2 Gene enrichment analysis of *HTRA1* and coregulated genes

**FIGURE 2:**
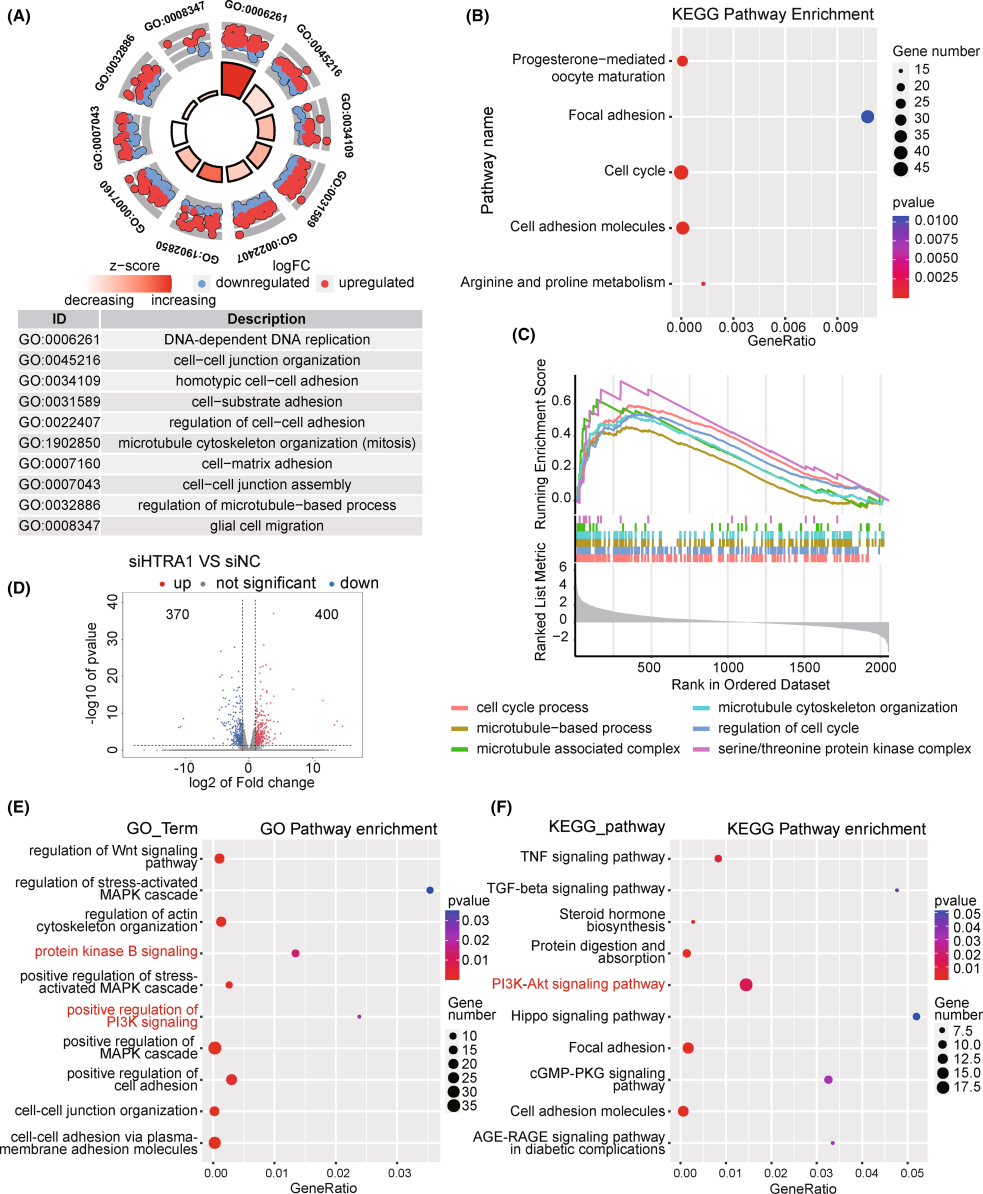
Gene enrichment analysis of HTRA1 coregulated genes. (A–C) Gene enrichment analysis of expression‐correlated genes of HTRA1 by (A) GO, (B) KEGG, and (C) GSEA enrichment analysis. (D) Volcano plot showing the DEGs derived from RNA‐seq analysis. (E) GO and (F) KEGG enrichment analysis of DEGs derived from RNA‐seq analysis.

Enrichment analysis was performed for genes with expression levels that were significantly correlated with *HTRA1* (*r* > 0.3 or *r* < −0.3) to identify potential signaling pathways and biological processes involving the gene. GO (Figure 2A) and KEGG (Figure 2B) analyses and GSEA (Figure 2C) yielded enriched pathways related to the cell cycle, cell adhesion and microtubule‐based processes in. These results indicated that HTRA1 might participate in cell proliferation and cell adhesion processes. We also performed RNA‐seq analysis of GBM#P3‐shNC and ‐shHTRA1 cells to identify putative genes regulated by HTRA1. Differentially expressed genes included 400 upregulated and 370 downregulated genes in the GBM#P3‐shHTRA1 cells compared with GBM#P3‐shNC cells (Figure 2D). In GO and KEGG enrichment analyses of the differentially expressed genes, cell adhesion‐related pathways were among the top 10 identified pathways (Figure 2E,F). This result was in agreement with the previous analysis. In addition, pathways related to PI3K‐Akt, Wnt and TGF‐β signaling pathways were also identified, and PI3K‐Akt‐related pathways were found both in top 10 of GO and KEGG enrichment analysis. However, cell cycle‐related pathways were not among the top 20 pathways. Overall, we concluded that *HTRA1* and coregulated genes might be involved in the regulation of cell migration and microtubule‐based processes.

### 3.3 HTRA1 knockdown suppresses proliferation and causes apoptosis in GBM cells

**FIGURE 3:**
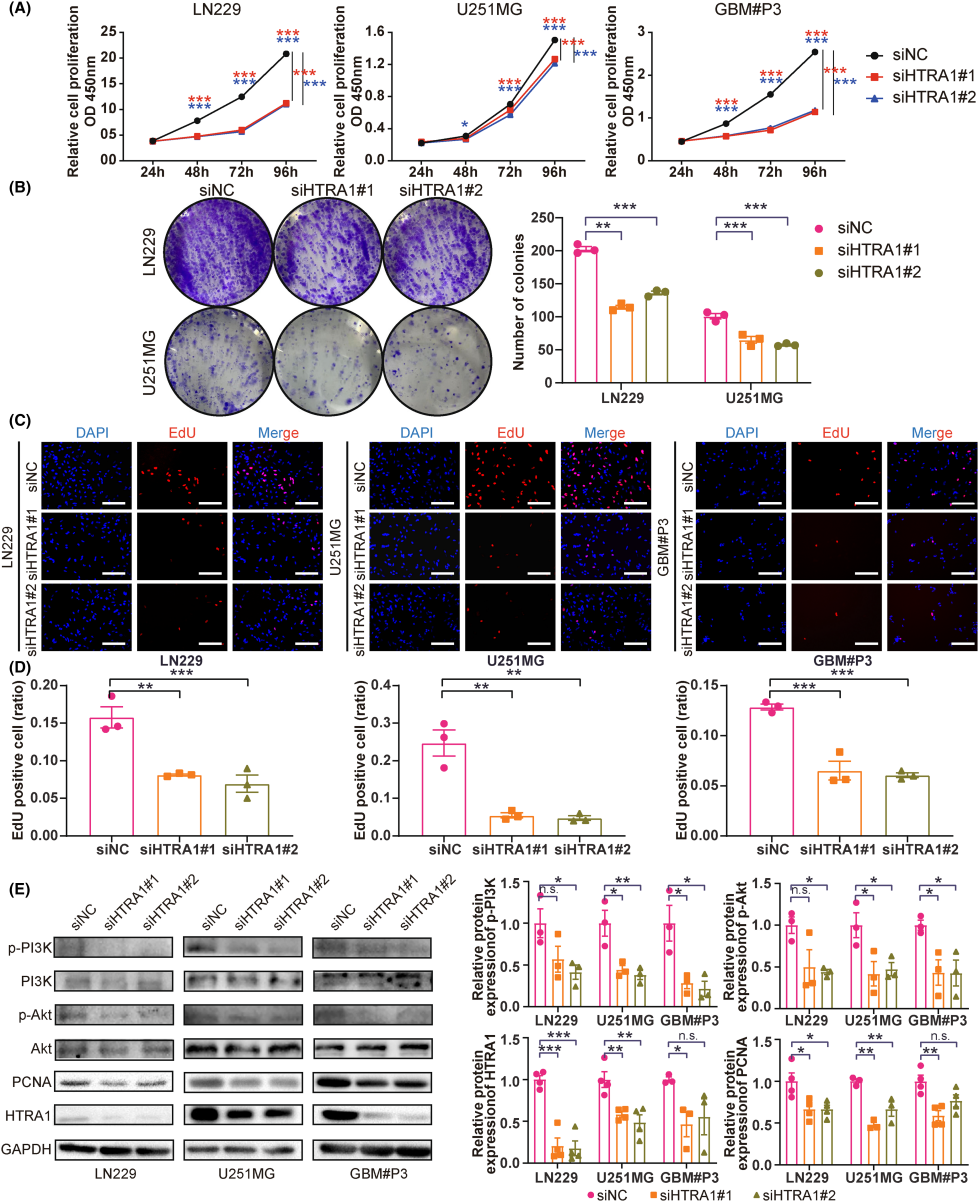
Downregulation of HTRA1 suppresses the growth of GBM cells. (A) CCK‐8 assays detecting the cell viabilities of LN229, U251MG and GBM#P3 cells transfected with siNC, siHTRA1#1 and siHTRA1#2. (B) Representative images of colony formation assay and colony number counts of LN229 and U251MG cells treated with HTRA1 siRNA. (C) Representative images and (D) positive ratios for EdU assays performed on LN229, U251MG and GBM#P3 cells with knockdown of HTRA1. Bar = 100 μm. (E) Western blots showing knockdown efficiency of HTRA1 siRNAs and levels of PI3K‐Akt pathway proteins (p‐PI3K, PI3K, p‐Akt, Akt), as well as proliferation marker (PCNA) in LN229, U251MG and GBM#P3 cells. GAPDH was used as the control for loading. (A), two‐way ANOVA, post hoc Dunnett's test; (B, D and E), one‐way ANOVA, post hoc Dunnett's test. *P < 0.05; **P < 0.01; ***P < 0.001.

The results of the enrichment analysis indicated that *HTRA1* and co‐expressed genes are involved in the cell cycle, which is tightly correlated with cell proliferation. Therefore, we examined the proliferation of GBM cells with siRNA knockdown of HTRA1 via CCK8 assay. Loss of HTRA1 induced a statistically significant reduction in relative cell proliferation values after 48 h in LN229, U251MG and GBM#P3 cells (Figure 3A). In colony formation assays, the number of colonies was reduced in cells transfected with HTRA1 siRNA (Figure 3B). LN229, U251MG and GBM#P3 cells also exhibited decreased EdU‐positive ratios after HTRA1 knockdown, which suggested a reduced proliferative cell ratio (Figure 3C,D). Furthermore, the cell proliferation marker PCNA^33^ was also decreased, as assessed on western blots, and the results suggested that the PCNA levels significantly decreased after downregulating HTRA1 in LN229, U251MG and GBM#P3 cells (Figure 3E). Besides, according to the enrichment analyses mentioned above (Figure 2E,F), we analyzed PI3K‐Akt pathway in GBM cells with HTRA1 knockdown, which was tightly associated with cell proliferation.^34^ The results showed that the levels of activated forms of PI3K and Akt decreased significantly after HTRA1 knockdown (Figure 3E). The effect of HTRA1 on cell cycle was also examined via flow cytometry. And the results indicated HTRA1 knockdown could lead to an increase in the proportion of cells in G0/G1 phase, and the decreases in G2/M proportion suggested that cell proliferation and division was inhibited (Figure S5). These data suggested that HTRA1 might promote GBM cell proliferation.

**FIGURE 4:**
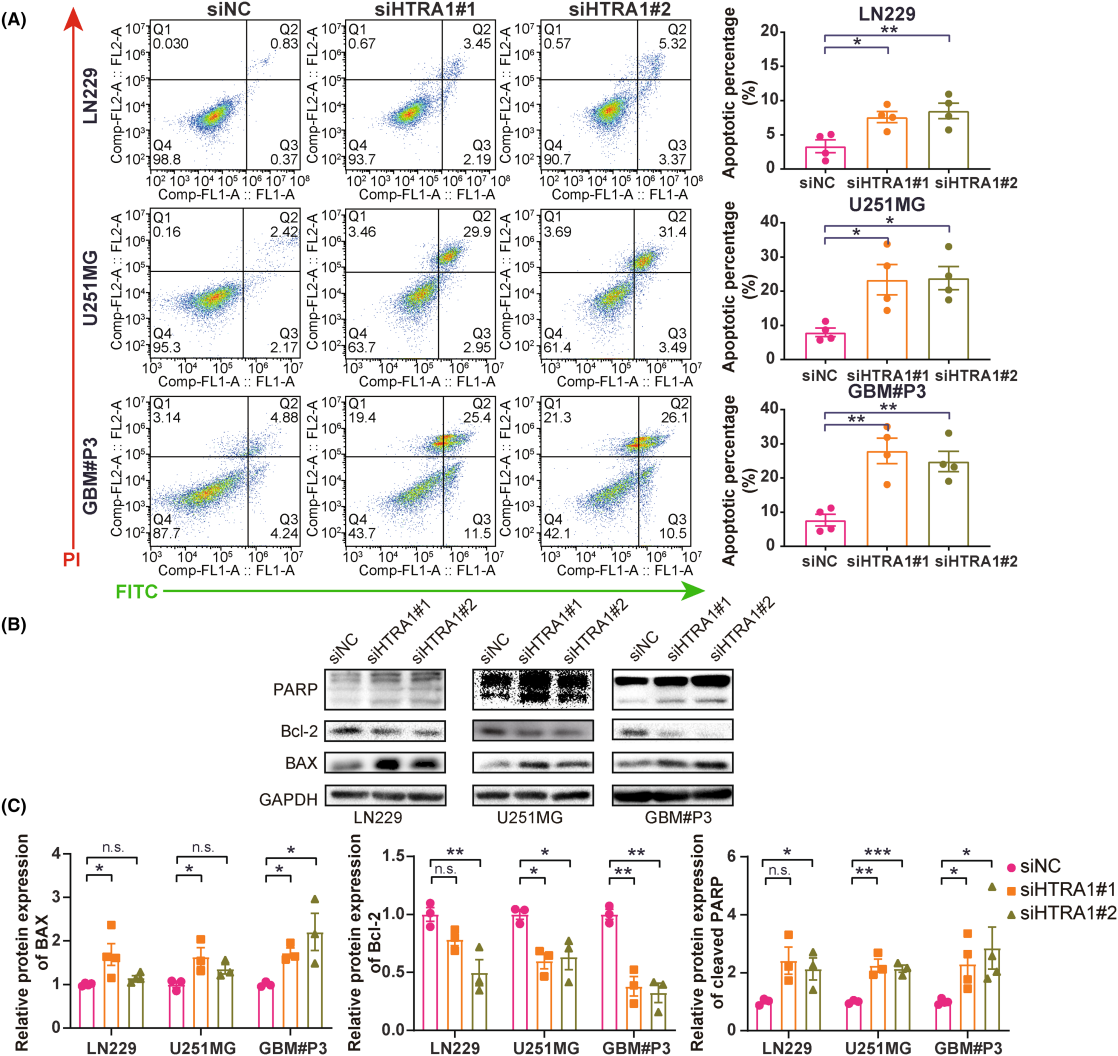
HTRA1 knockdown promotes apoptosis of GBM cells. (A) Flow cytometry analysis detecting percentage apoptosis of LN229, U251MG and GBM#P3 cells transfected with siNC, siHTRA1#1 and siHTRA1#2. (B) Representative images of western blots and (C) relative protein levels of apoptosis‐related proteins in lysates prepared from LN229, U251MG and GBM#P3 cells with HTRA1 knockdown. (A, C), one‐way ANOVA, post hoc Dunnett's test. *P < 0.05; **P < 0.01; ***P < 0.001.

Under evaluation with microscopy, we frequently found more apoptotic cells after HTRA1 knockdown. We, therefore, determined the ratio of apoptotic cells with flow cytometry. The ratios of apoptotic cells in LN229, U251MG and GBM#P3 transfected with HTRA1 siRNA significantly increased compared with cells transfected with siNC (Figure 4A). Moreover, we found protein levels of apoptosis markers cleaved PARP and BAX^35^ increased while an apoptosis inhibitor BCL2 decreased^35^ in LN229, U251MG and GBM#P3 with knockdown of HTRA1 (Figure 4B,C). Overall, we found that HTRA1 might be involved in promoting proliferation and inhibiting apoptosis in GBM cell lines.

### 3.4 HTRA1 knockdown inhibits the migration and invasion of GBM cells

**FIGURE 5:**
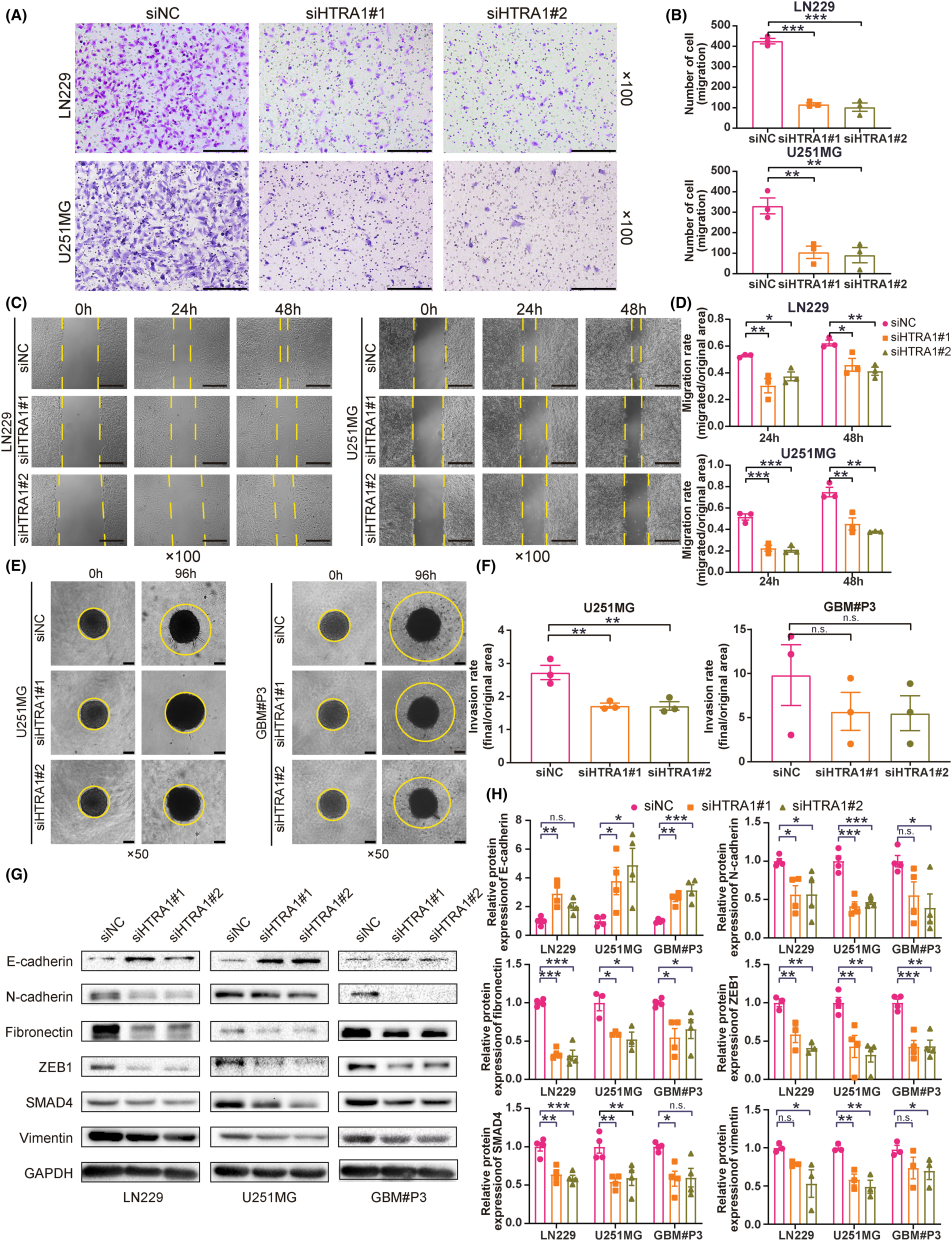
HTRA1 knockdown inhibits cell migration and proliferation of GBM cells in vitro. (A, B) Representative images of transwell assays and cell counting results for LN229 and U251MG cells transfected with HTRA1 siRNAs. Bar = 200 μm. (C, D) Representative images of wound healing assay and migration rates for LN229 and U251MG cells transfected with HTRA1 siRNAs. Bar = 200 μm. (E, F) Representative images of 3D invasion assays and invasion rates for U251MG and GBM#P3 cells transfected with HTRA1 siRNAs. Bar = 100 μm. (G, H) Representative images of western blots and relative expression levels of EMT‐related proteins in lysates prepared from LN229, U251MG and GBM#P3 cells transfected with HTRA1 siRNA. (B, D, F and H), one‐way ANOVA, post hoc Dunnett's test. *P < 0.05; **P < 0.01; ***P < 0.001.

The enrichment analysis indicated a putative role for HTRA1 in cell adhesion processes. We, therefore, investigated whether HTRA1 was involved in the migration and invasion of GBM cells. First, transwell assays revealed a significant decrease in the number of migrated cells per view after knockdown of HTRA1 in LN229 and U251MG cells (Figure 5A,B). Second, LN229 and U251MG cells transfected with HTRA1 siRNA also showed a decreased migration rate in the wound healing assay (Figure 5C,D). These results indicated that the migration ability of GBM cells decreased after HTRA1 knockdown. Finally, in the 3D spheroid invasion assay, we found that the U251MG and GBM#P3 spheroids with loss of HTRA1 exhibited a significantly decreased invasion area compared to the siNC spheroid controls (Figure 5E,F). These indicated that HTRA1 knockdown decreased the invasion ability of GBM cells.

It is well‐known that epithelial‐mesenchymal transition (EMT) plays a crucial role in the migration and invasion of tumor cells.^36^, ^37^ We, therefore, investigated whether HTRA1 was involved in EMT of GBM cells. We found that expression of mesenchymal markers, including N‐cadherin, fibronectin, SMAD4 and vimentin, as did that of the upstream transcription factor ZEB1, significantly decreased with HTRA1 knockdown (Figure 5G,H). In contrast, the expression of the epithelial marker E‐cadherin increased with HTRA1 knockdown (Figure 5G,H). These results indicated that HTRA1 promoted EMT in GBM cells.

### 3.5 HTRA1 facilitates the formation of invadopodia and destabilizes microtubules via HDAC6‐mediated deacetylation

**FIGURE 6:**
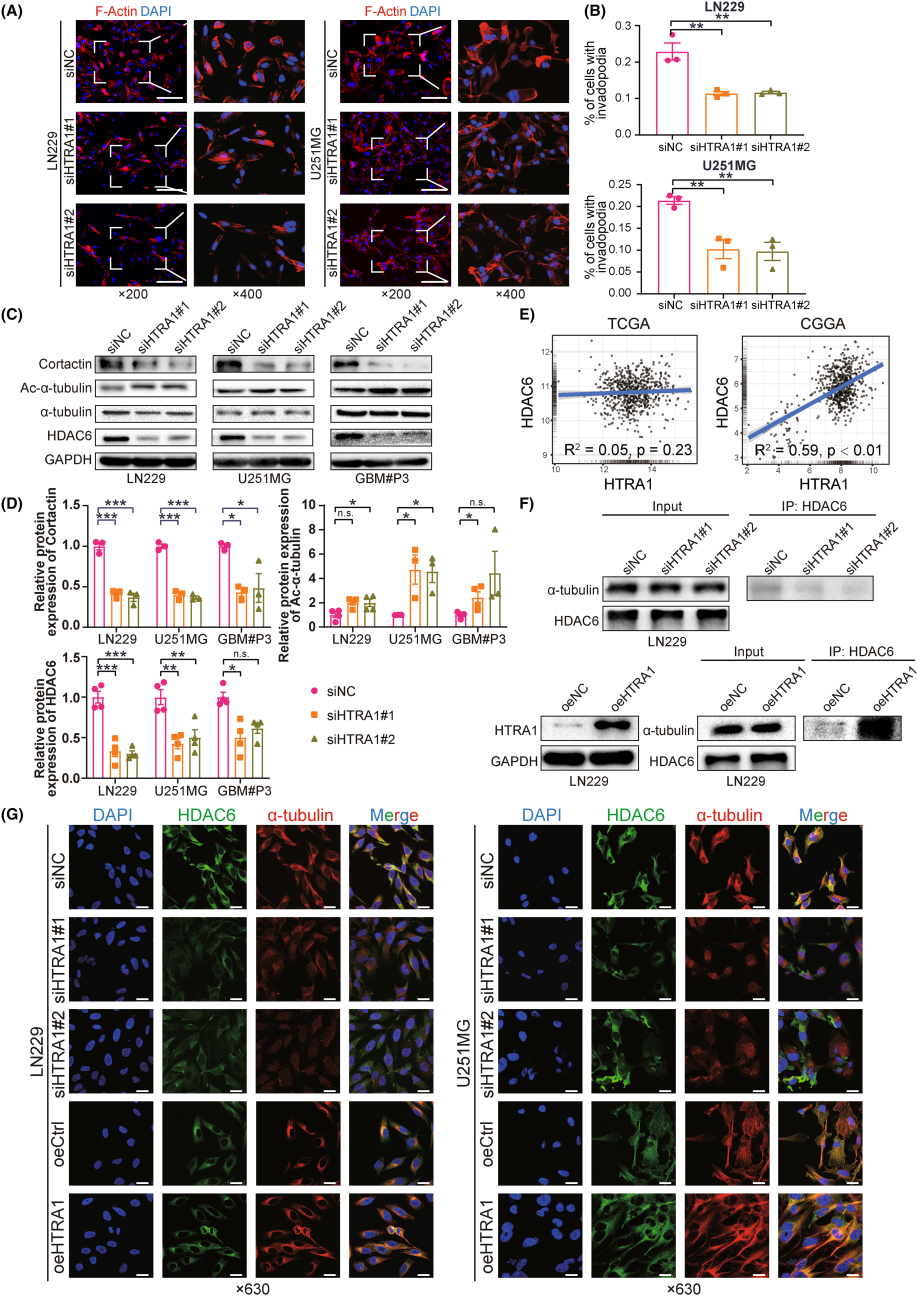
Knockdown of HTRA1 inhibits formation of invadopodia in glioma cells via the HDAC6/Ac‐α‐tubulin pathway. (A, B) Representative fluorescence images of staining of invadopodia and formation rates in LN229 and U251MG cells transfected with HTRA1 siRNAs. Bar = 100 μm. (C, D) Western blots for the detection of Cortactin, HDAC6, α‐tubulin and ac‐α‐tubulin in LN229, U251MG and GBM#P3 cells transfected with HTRA1 siRNA. (E) Correlation analyses for mRNA expression levels of *HTRA1* and *HDAC6* based on TCGA and CGGA databases. (F) Western blots of lysates prepared from LN229‐siHTRA1 or oeHTRA1 cells (left panel) and immunoprecipitations (right panels) performed with anti‐HDAC6 antibody showing binding of HDAC6 to α‐tubulin in the presence of increased HTRA1. (G) Representative images of immunofluorescence detecting co‐localization between HDAC6 and α‐tubulin in LN229 and U251MG cells transfected with HTRA1 siRNAs or HTRA1 overexpression plasmids. Bar = 25 μm. (B, D), one‐way ANOVA, post hoc Dunnett's test; for ac‐α‐tubulin of LN229 and HDAC6 of GBM#P3 in (D), Kruskal‐Wallis, post hoc Dunn's test. *P < 0.05; **P < 0.01; ***P < 0.001.

To further investigate whether HTRA1 promoted invasion and migration, we evaluated cytoskeleton‐ and microtubule‐based processes in GBM cells transfected with HTRA1 siRNA. We first used phalloidin detection of F‐actin, a protein involved in the formation of invadopodia and cell migration.^38^ With knockdown of HTRA1, the percentage of LN229 and U251MG cells with invadopodia significantly decreased (Figure 6A,B). And the expression levels of cortactin, a marker of invadopodia formation,^39^ declined after HTRA1 knockdown (Figure 6C,D). These results were consistent with the decreased migration ability observed in wound healing assays (Figure 5C,D). Second, the formation of invadopodia is also previously reported to be correlated with cytoskeleton formation regulated by HDAC6, a deacetylase that causes the deacetylation of α‐tubulin.^40^ The increase in ac‐α‐tubulin levels has also been reported to be associated with inhibition of migration and invasion.^41^ Our results showed that the protein levels of HDAC6 were decreased, while the protein levels of ac‐α‐tubulin were significantly increased in GBM cells with HTRA1 knockdown (Figure 6C,D). Third, *HTRA1* and *HDAC6* mRNA levels were positively correlated in the TCGA and CGGA databases (Figure 6E). Besides, immunoprecipitations performed on lysates prepared from LN229‐siHTRA1 cells demonstrated that the interaction between HDAC6 and α‐tubulin was decreased with the knockdown of HTRA1 in GBM cells, and samples with HTRA1 overexpression exhibited reverse results (Figure 6F). Last but not the least, the immunofluorescence detection indicated that the co‐localization of HDAC6 and α‐tubulin was suppressed after downregulation of HTRA1 in GBM cells, and overexpression of HTRA1 enhanced the HDAC6/α‐tubulin co‐localization (Figure 6G).

These results suggested that HTRA1 promoted cell migration by decreasing ac‐α‐tubulin levels. Thus, increased HTRA1 expression was associated with increased HDAC6 expression, and the HDAC6‐α‐tubulin interaction was enhanced with overexpression of HTRA1. These results were consistent with the presence of an HDAC6/ac‐α‐tubulin axis in GBM cells.

### 3.6 Knockdown of HTRA1 inhibits GBM cell growth in vivo and prolongs overall survival of tumor‐bearing mice

**FIGURE 7:**
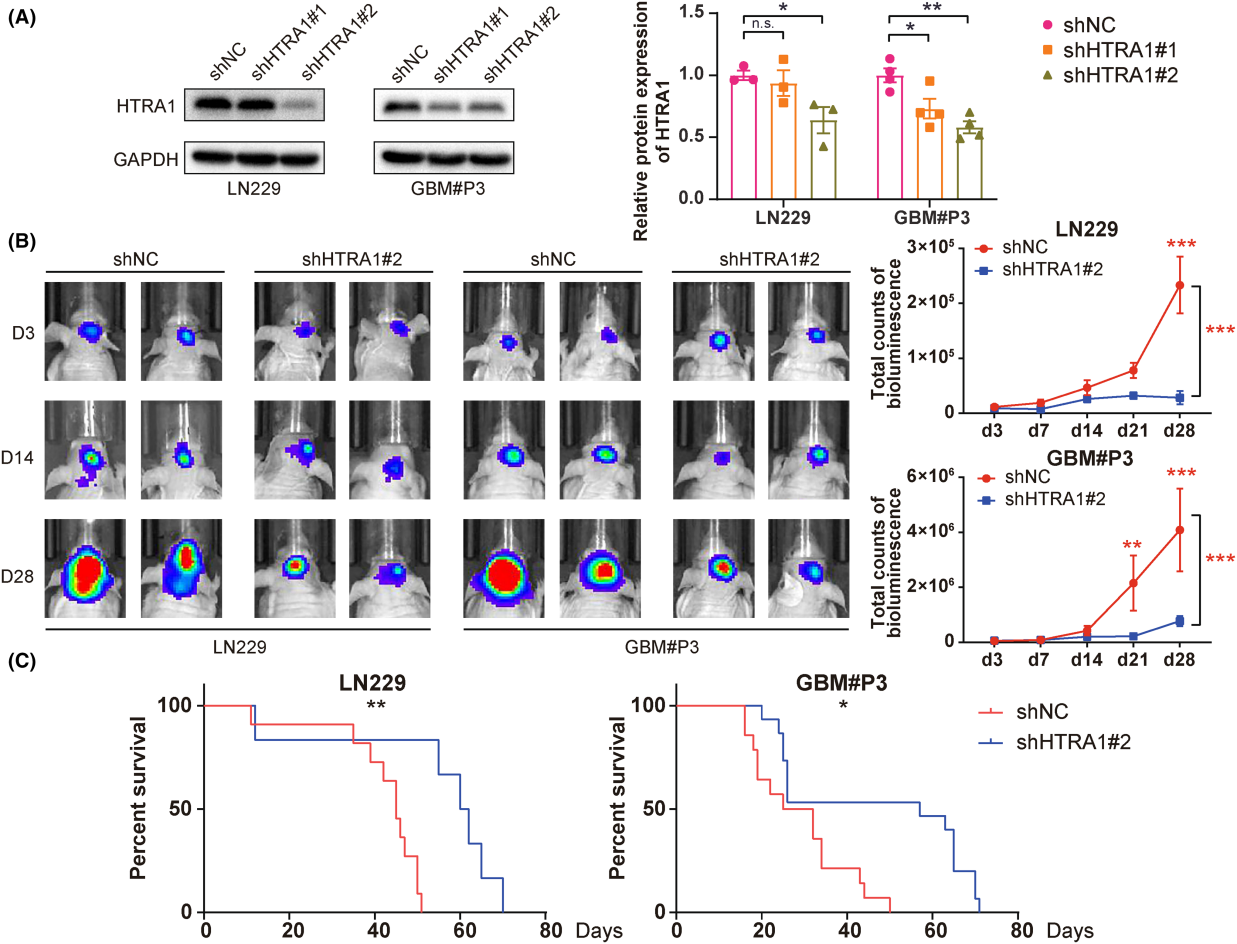
Downregulation of HTRA1 inhibits GBM cell growth in vivo. (A) Western blot results indicating that shHTRA1#2 works well in both LN229 and GBM#P3 cells. (B) Representative images of in vivo tumor imaging and total bioluminescence from day 3 to day 28. (C) Survival rates of LN229‐ and GBM#P3‐shHTRA1 tumor‐bearing mice. (A), one‐way ANOVA, post hoc Dunnett's test; (B), two‐way ANOVA, post hoc Dunnett's test; (C), log‐rank test. *P < 0.05; **P < 0.01; ***P < 0.001.

**FIGURE 8:**
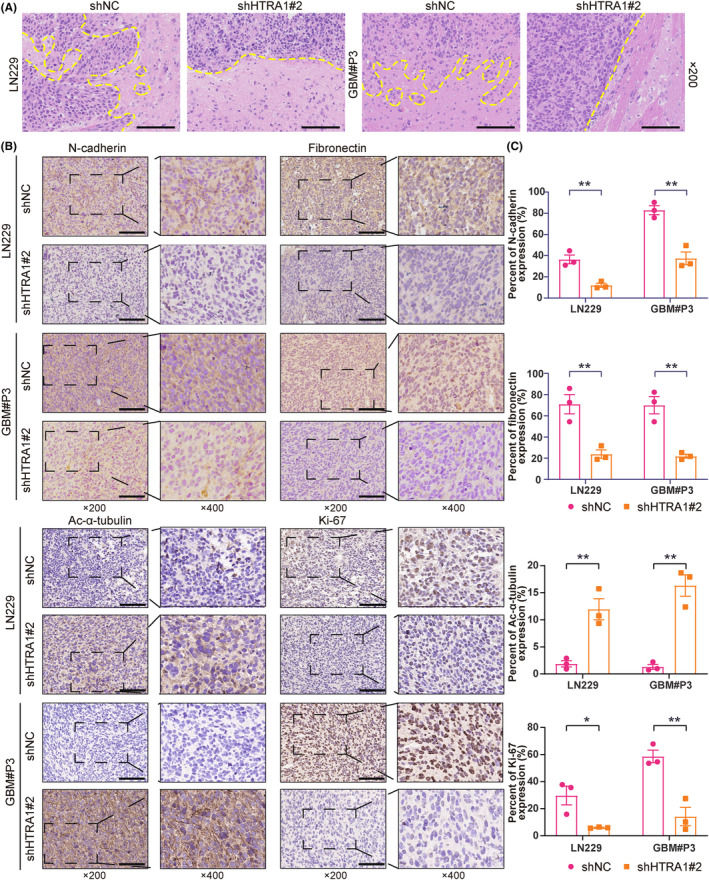
Histologic results showing knockdown of HTRA1 downregulates GBM cell invasion and growth in vivo. (A) Representative images of HE staining showing the margins between xenografts and normal brain. Bar = 100 μm. (B, C) Representative images of immunohistochemistry detecting N‐cadherin, fibronectin, ac‐α‐tubulin and Ki‐67 in LN229‐ and GBM#P3‐shHTRA1 xenografts and the percent of expression of these four proteins. (C), Student's *t*‐test. *P < 0.05; **P < 0.01. Bar = 100 μm.

To study the effect of HTRA1 on in vivo tumor growth, we stably knocked down HTRA1 in LN229 and GBM#P3 cells expressing luciferase. LN229 and GBM#P3 cells expressing luciferase were transduced with lentivirus containing siRNA sequences, and the knockdown efficiency was assessed on western blot (Figure 7A). The results indicated that only shHTRA1#2 efficiently knocked down HTRA1 in both LN229 and GBM#P3 cells. LN229^luc^‐ and GBM#P3^luc^‐shHTRA1#2 cells (and controls, ‐shNC) were implanted into the cortex of nude mice brains, and the tumor size was assessed through measurement in vivo bioluminescence at 3, 7, 14, 21 and 28 days after orthotopic implantation. The bioluminescence of both LN229^luc^‐ and GBM#P3^luc^‐shHTRA1#2 intracranial tumors was significantly reduced at 21 to 28 days after implantation relative to controls (Figure 7B). In addition, the overall survival of tumor‐bearing animals was significantly longer for the shHTRA1 group than for the shNC group (Figure 7C). In terms of histology, the results of HE staining showed suppressed invasion ability at the margin of xenograft tumor in LN229^luc^‐ and GBM#P3^luc^‐shHTRA1#2 group (Figure 8A). IHC revealed decreased expression of mesenchymal markers, including N‐cadherin and fibronectin, as well as cell proliferation marker Ki‐67, in LN229^luc^‐ and GBM#P3^luc^‐shHTRA1#2 xenografts relative to shNC controls (Figure 8B,C). In contrast, the number of cells positive for ac‐α‐tubulin was significantly increased in LN229^luc^‐ and GBM#P3^luc^‐shHTRA1#2 xenografts relative to shNC controls. These in vivo results confirmed our in vitro results. Taken together, the downregulation of HTRA1 in vivo resulted in suppressed GBM cell proliferation, but increased ac‐α‐tubulin levels.

## 4 DISCUSSION

In this study, we found increased HTRA1 expression in gliomas relative to nontumor brain tissue. High HTRA1 expression was associated with the more aggressive glioma molecular subtypes, mesenchymal and classical subtypes. GO and KEGG analysis of HTRA1 coregulated genes revealed enrichment of several pathways related to cell migration and invasion, and knockdown of HTRA1 with siRNA led to reduced migration, invasion and invadopodia formation in GBM cells. HTRA1 knockdown also reduced proliferation but increased apoptosis in cells. Analysis of transcriptome sequencing data from LN229‐ and GBM#P3‐shHTRA1#2 revealed that differentially expressed genes were enriched in pathways related to cell adhesion and migration. In addition, transcription factor analysis demonstrated that the transcription factor SMAD4 might be involved in these gene expression changes often associated with EMT in tumor cells.^42^ In vivo orthotopic implantation of modified GBM cells indicated that HTRA1 knockdown inhibited intracranial tumor growth and prolonged overall survival of nude mice.

Glioma is an intracranial tumor type resistant to the current standard of care, and invasion and migration are key properties contributing to this resistance. EMT is an essential and dynamic process driving the invasion and migration of tumor cells.^43^, ^44^ Here, we report that HTRA1 depletion significantly inhibited the migration and invasion abilities of GBM cells, and mesenchymal markers, including ZEB1, N‐cadherin, vimentin, fibronectin and CD44, decreased, while the epithelial marker E‐cadherin increased. Changes in the F‐actin cytoskeleton was consistent with the suppression of the formation of invadopodia, which are protrusions of the cell membrane that are rich in F‐actin and facilitate the dissemination of tumor cells.^45^ Previous reports indicate that HDAC6 is involved in cytoskeletal organization as well as invadopodia formation through deacetylation of α‐tubulin.^40^ HDAC6 is a member of the HDAC family that is mainly located in the cytoplasm and promotes tumor cell migration and invasion through deacetylation of α‐tubulin and cortactin, which destabilizes the cytoskeleton.^46^ HDAC6 expression decreased in GBM cells with HTRA1 depletion, while ac‐α‐tubulin increased. These results indicated that HTRA1 depletion led to decreased migration and invasion by enhancing the stability of the cytoskeleton. We also observed an increase of interaction between HDAC6 and α‐tubulin when HTRA1 was over expressed, but the activity of HTRA1 on HDAC6 requires further investigation.

HTRA1 has been reported to promote apoptosis in many types of tumor cells, such as esophageal squamous cell carcinoma^47^ and pancreatic cancer cells.^48^ Our study demonstrated that apoptosis was enhanced in GBM cells with depletion of HTRA1, and markers of apoptosis were also correspondingly regulated. These results indicated that HTRA1 inhibited apoptosis in human GBM cells.

Our findings are consistent with a role for HTRA1 as an oncogene in GBM cells. These results support a previous study on gastric cancer,^12^ in which HTRA1 was reported to induce the transdifferentiation of fibroblasts into cancer‐associated fibroblasts by activating the NF‐κB/bFGF pathway. In addition, HTRA1 was recently reported to promote tumor metastasis by binding to the pro‐oncogenic protein PITPNC1.^49^ On the other hand, HTRA1 is widely regarded as a tumor suppressor. HTRA1 was shown to suppress the proliferation of tumor cells by inhibiting Wnt/β‐catenin through complexing with β‐catenin.^11^ Moreover, HTRA1 was reported to suppress angiogenesis in cancer stromal cells by activating the Notch pathway.^7^

A limitation of this study is that we did not‐test any HTRA1 inhibitor in vivo to explore its translational utility. Most of the known inhibitors, such as galegenimab,^50^ 6‐boroV^51^ and NVP‐LBG976^52^ were reported as peptide inhibitors so it would be hard for them to pass through brain–blood barrier. Thus, it is likely that small molecules that inhibit HTRA1 may be beneficial for glioma treatment in vivo and further studies are required for confirmation.

In summary, the role of HTRA1 as an oncogene or tumor suppressor might be dependent on cellular context. Our work indicates that HTRA1 might promote the development of human glioma by enhancing cell migration and invasion. However, the molecular mechanisms underlying HTRA1 activity in glioma requires further investigation.

## 5 CONCLUSION

Our current research provides evidences that HTRA1 might be a carcinogenic gene in glioma cells. HTRA1 also promoted migration of glioma cells. This effect might be mediated via downregulating acetylated α‐tubulin by facilitating HDAC6 expression. Our work explores the role of HTRA1 in glioma cells and also provides new insights for the infiltration of glioma cells.

## AUTHOR CONTRIBUTIONS

Wenbo Zhao conducted all experiments. Shuai Wang collected and analyzed data. Feihu Zhao and Wenyu Liu helped to conduct animal experiments. Zhiyi Xue, Lin Zhang, Jian Wang and Mingzhi Han wrote the manuscript. Xingang Li and Bin Huang designed all experiments.

## CONFLICT OF INTEREST STATEMENT

The authors declare no conflict of interest.

## Supporting information


Figure S1.

Figure S2.

Figure S3.

Figure S4.

Figure S5.

Data S1.

## Data Availability

Data are available upon reasonable request from the corresponding author.
