# Supplementary figures and images for: HTRA1 promotes EMT through the HDAC6/Ac‐α‐tubulin pathway in human GBM cells
Source: CNS Neurosci Ther. 2024 Feb 9;30(2):e14605. doi: 10.1111/cns.14605 (PMC10853898; doi:10.1111/cns.14605)

**(A)**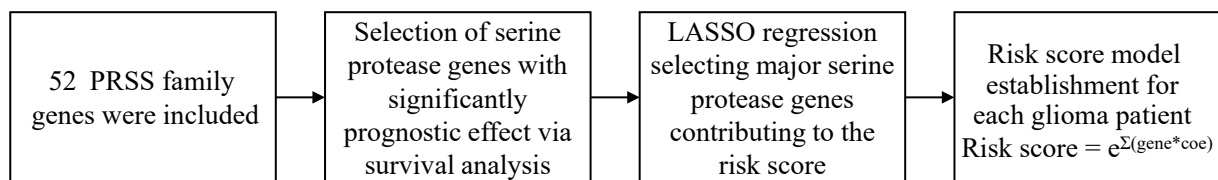**(B)**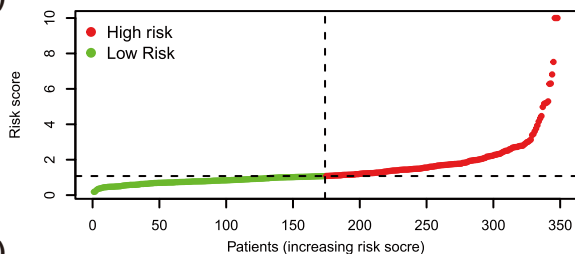**(C)**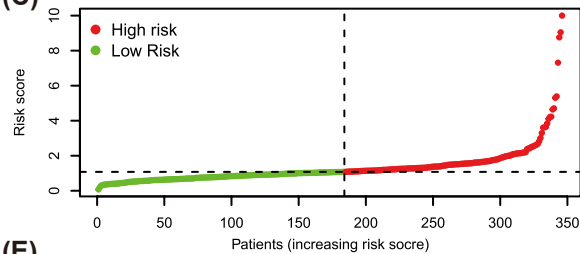**(D)**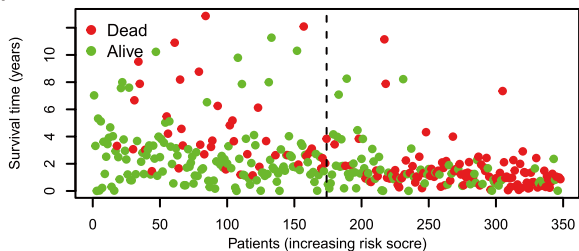**(E)**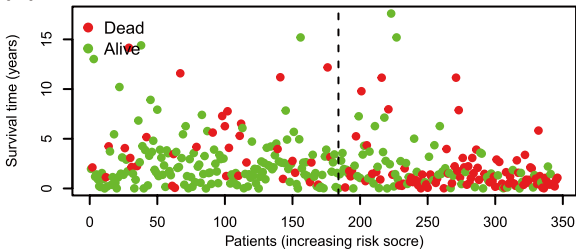**(F)**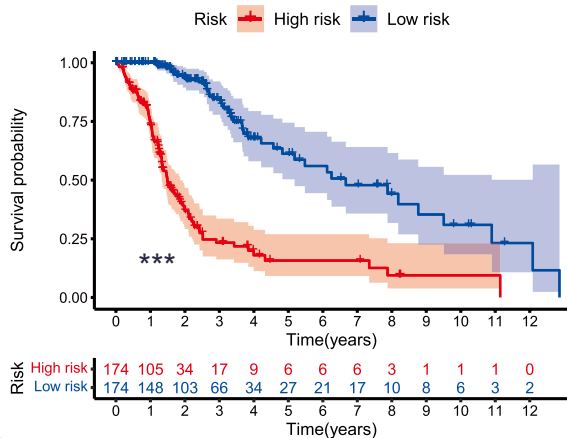**(G)**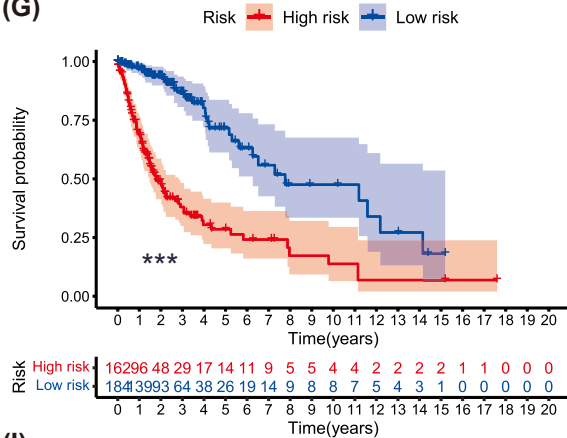**(H)**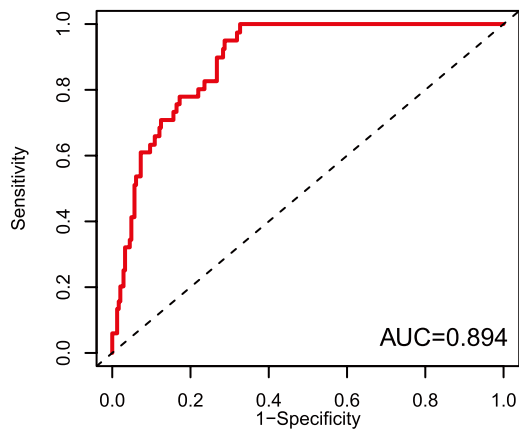**(I)**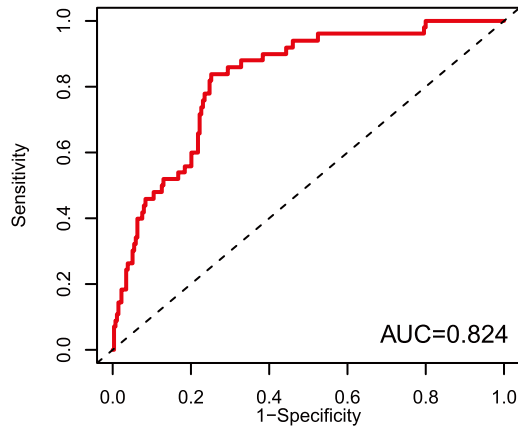

Supplement: Supplementary file 1 — Figure S1. [file CNS-30-e14605-s005.pdf]

**(A)**Risk 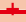 High risk 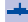 Low risk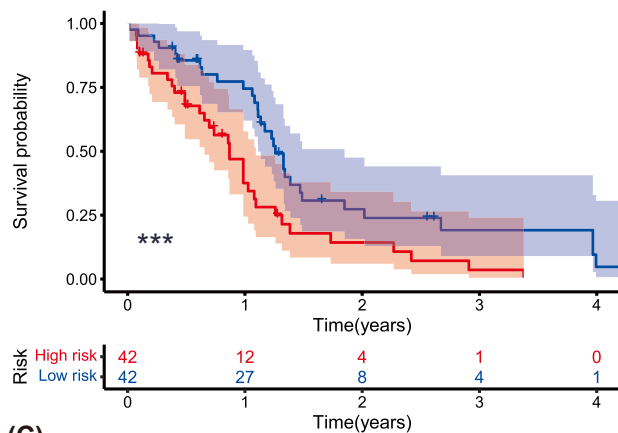**(B)**Risk 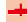 High risk 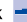 Low risk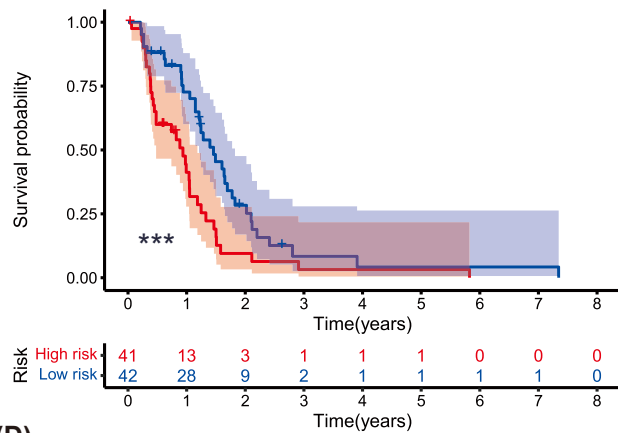**(C)**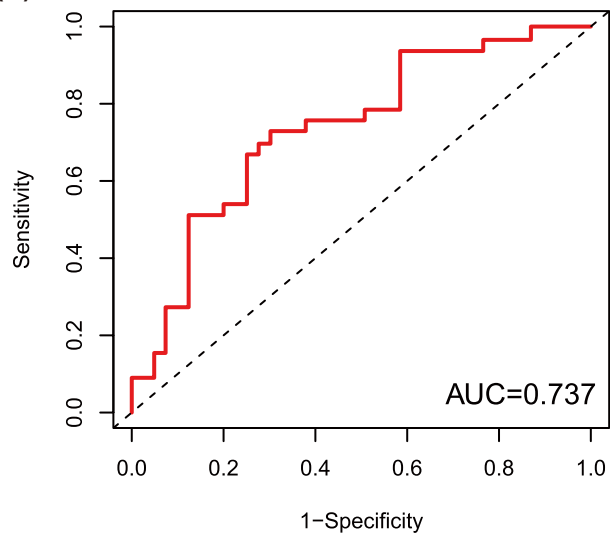**(D)**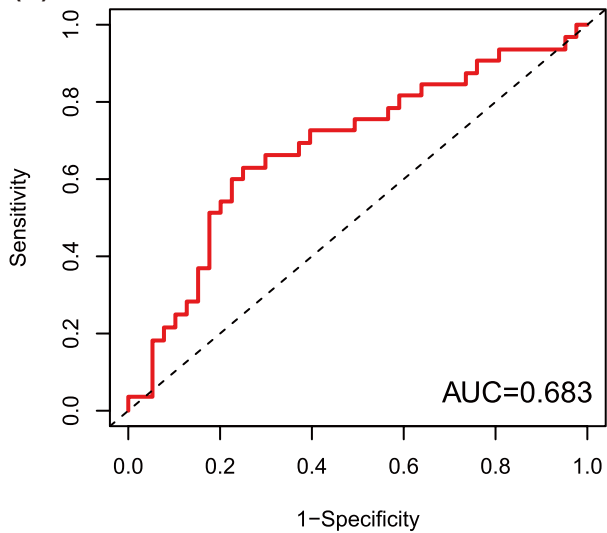

Supplement: Supplementary file 2 — Figure S2. [file CNS-30-e14605-s004.pdf]

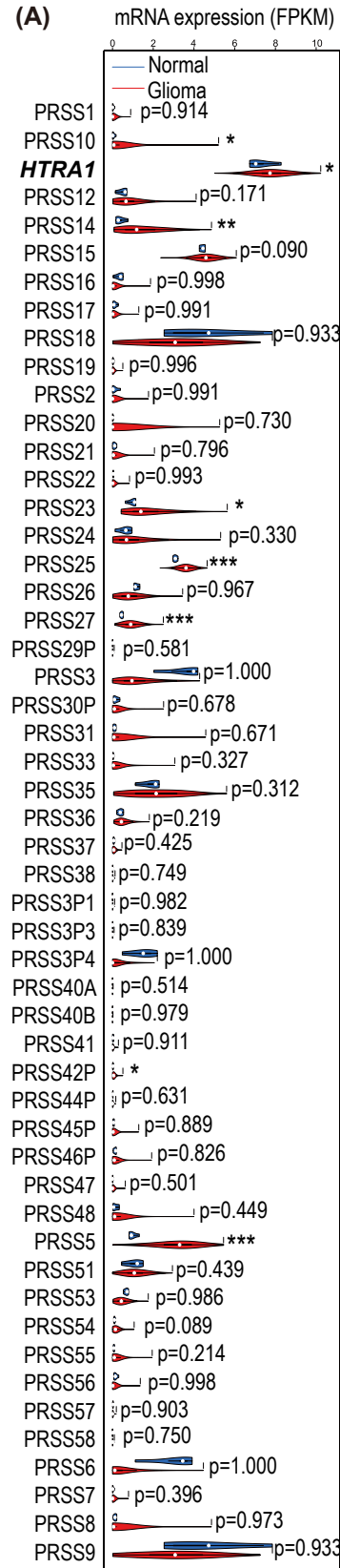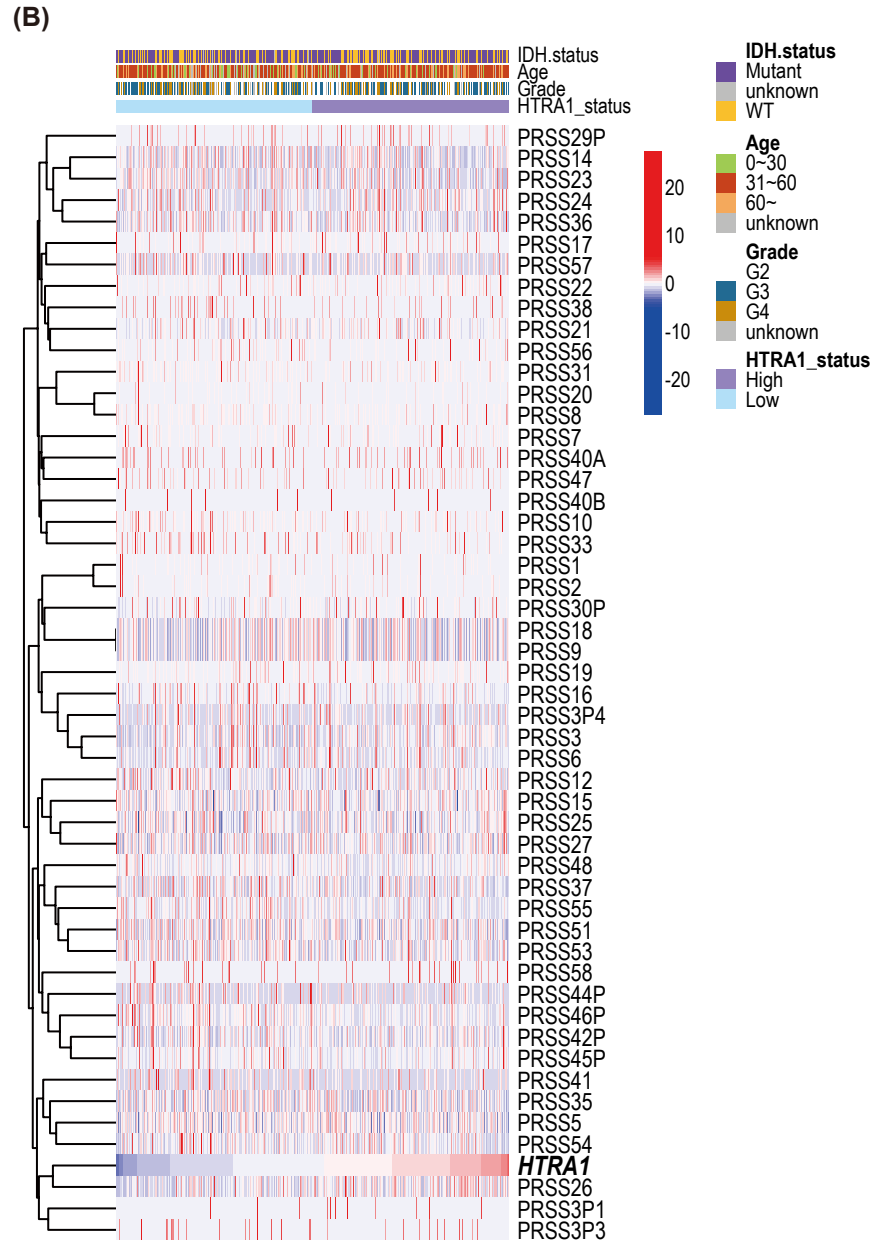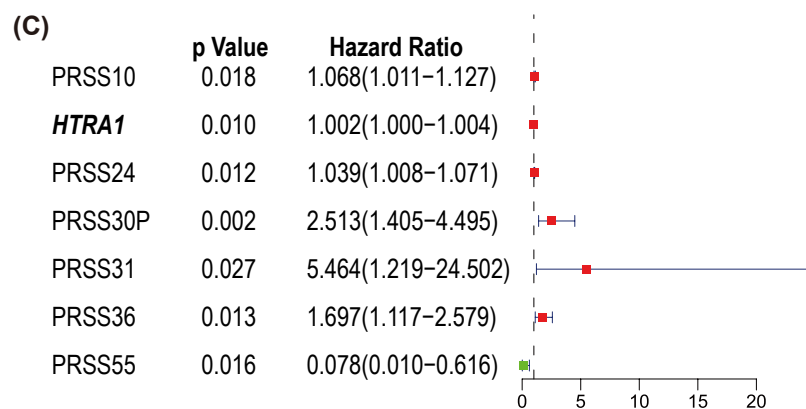

Supplement: Supplementary file 3 — Figure S3. [file CNS-30-e14605-s001.pdf]

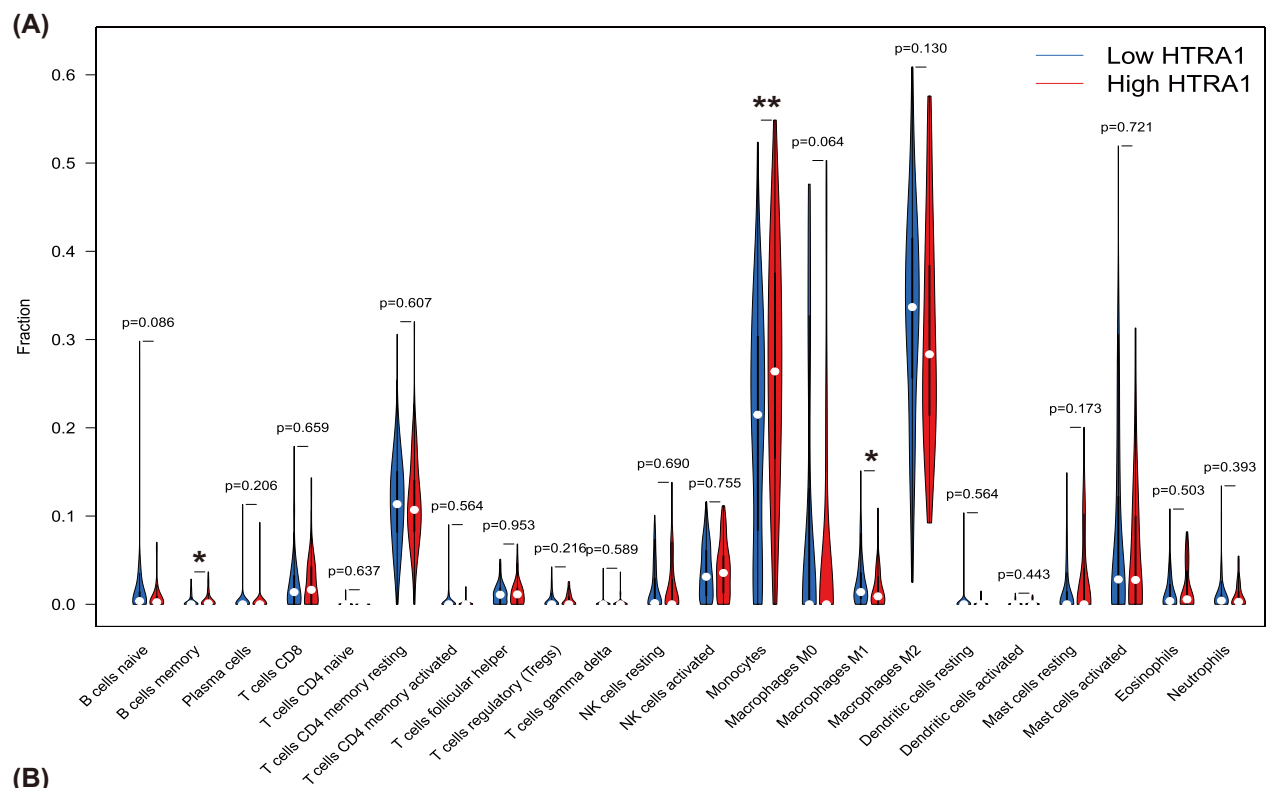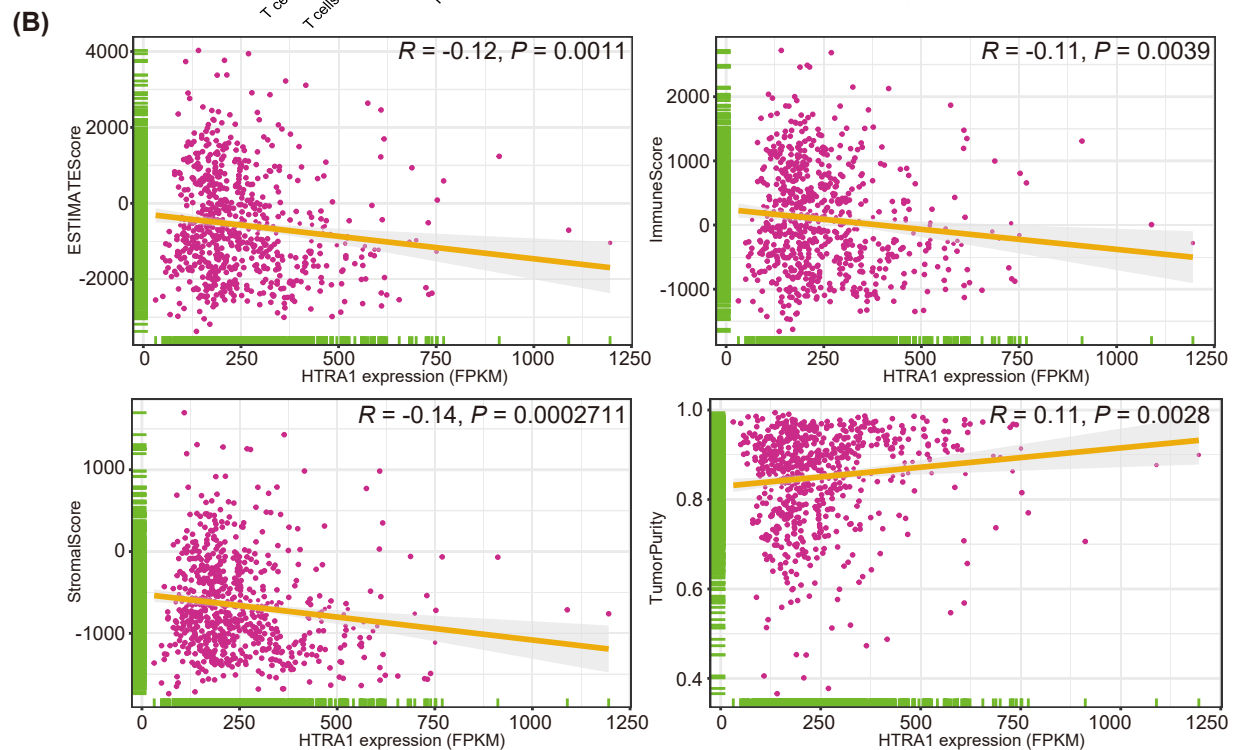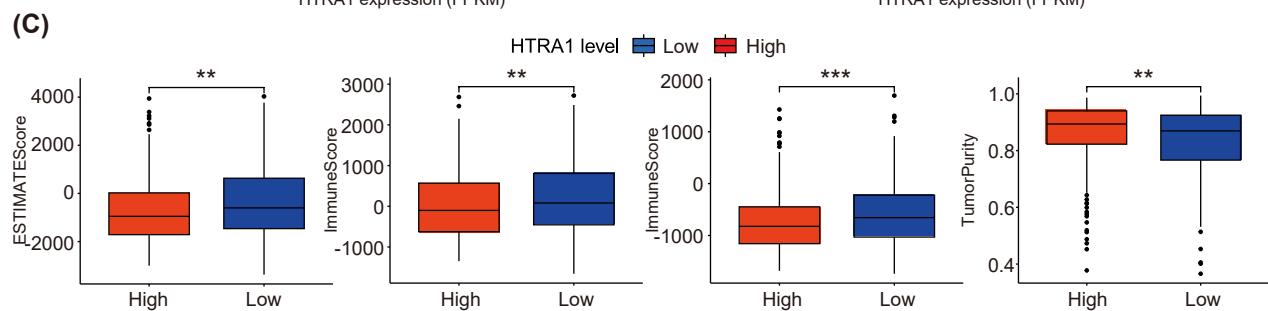

Supplement: Supplementary file 4 — Figure S4. [file CNS-30-e14605-s006.pdf]

**(A)**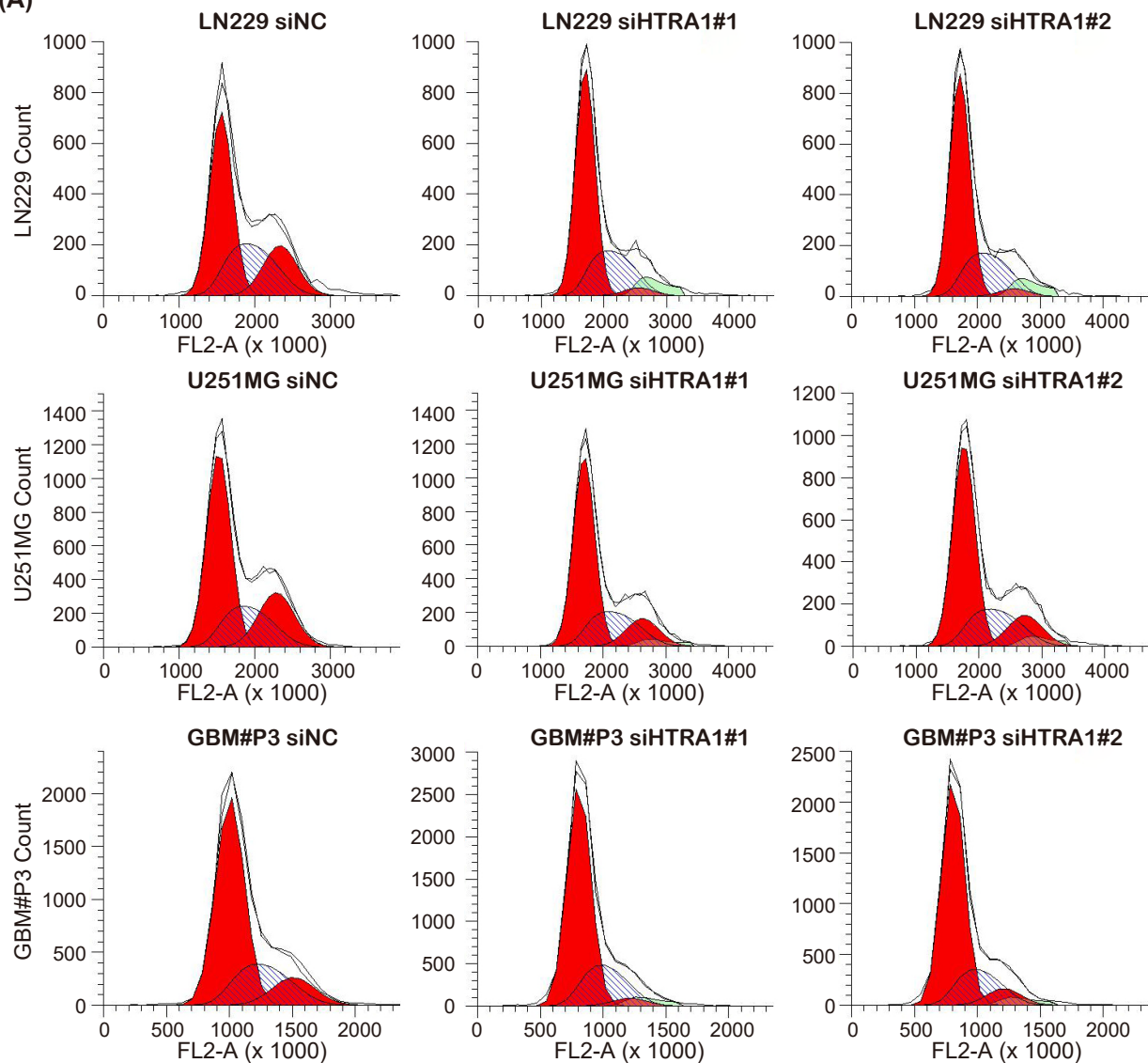**(B)**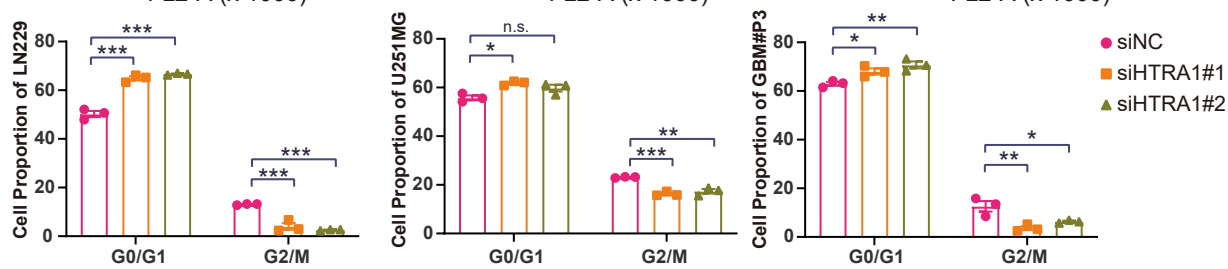

Supplement: Supplementary file 5 — Figure S5. [file CNS-30-e14605-s003.pdf]
