# Supplementary material for: HTRA1 promotes EMT through the HDAC6/Ac‐α‐tubulin pathway in human GBM cells
Source: CNS Neurosci Ther. 2024 Feb 9;30(2):e14605. doi: 10.1111/cns.14605 (PMC10853898; doi:10.1111/cns.14605)
Supplement: Supplementary file 6 — Data S1. [file CNS-30-e14605-s002.docx]

1. **MATERIALS AND METHODS**

**1.1 Immune microenvironment analysis**

The “ESTIMATE” package in R (version 4.1.3) was employed to assess the immune activity and tumor purity of glioma samples. The ratios of infiltration immunocytes were calculated with CIBERSORT algorithm in R (version 4.1.3).

**1.2 Gene ontology enrichment analysis**

The genes significantly correlated with HTRA1 at expression levels were obtained in GlioVis and subjected to GO, KEGG and GSEA pathway enrichment analyses. These analyses were conducted in R (version 4.1.3) with the package “clusterProfiler”.

**1.3 Cell culture**

LN229 and U251MG cells were purchased from the Culture Collection of the Chinese Academy of Sciences (Shanghai, China). GBM#P3 (mesenchymal subtype) cells derived from a primary GBM were a kind gift from Professor Rolf Bjerkvig (Department of Biomedicine, University of Bergen, Norway). LN229 and U251MG cells were cultured in Dulbecco’s modified Eagle’s medium (#D0822-500ML; Sigma-Aldrich; MO, USA) supplemented with 10% fetal bovine serum (#C04001-500; VivaCell; Denzlingen, Germany). GBM#P3 was cultured in neurobasal medium (#21103049; ThermoFisher Scientific; MA, USA) with 20 μL/mL B27 supplement (#17504044; ThermoFisher Scientific; MA, USA), 20 ng/mL epidermal growth factor (#PHG0311; ThermoFisher Scientific; MA, USA), and 10 ng/mL basic fibroblast growth factor (#100-18B; PeproTech; NJ, USA). Cells were kept in a humidified chamber in 5% CO_2_ at 37 °C.

**1.4 Immunohistochemistry and hematoxylin-eosin (HE) staining**

Gliomas of different grades and normal brain tissues were fixed in formalin, embedded in paraffin and sectioned (4 μm). After deparaffinization and rehydration, antigen retrieval was performed in sodium citrate buffer (pH 6.0) at 95 °C, and endogenous peroxidase was eliminated with 3% H_2_O_2_. Sections were blocked in 10% goat serum and incubated in primary antibody overnight at 4°C.Primary antibody against N-cadherin (#22018-1-AP, 1: 1, 000; Proteintech; IL, USA) or Fibronectin (#66042-1-Ig, 1: 1, 000; Proteintech; IL, USA) or acetyl-α-tubulin (#5335, 1: 200; Cell Signaling Technology; MA, USA) or Ki-67 (#GB111499, 1:400; Servicebio; Hubei, China) was applied. Primary antibodies were detected with a (3,3′-diaminobenzidine) DAB visualization kit (#ZLI-9017; ZSGB-BIO; Beijing, China) following the manufacturer’s instructions. The cell nucleus was stained with hematoxylin (#C0107; Beyotime Biotechnology; Shanghai, China), and images were acquired with an Olympus inverted microscope (Tokyo, Japan). HE staining was performed with HE staining kit under manufacturer’s instructions (#G1120, Solarbio; Beijing, China).

**1.5 RNA interference**

Small-interfering RNA (siRNA) targeting HTRA1 was transfected into cells as previously described.^1^ The sequences used were described in a previous study^2^ and are the following: #1: 5’ GAAGUGAUUGGAAUUAACATT3’ and #2: 5’ UGUUAAUUCCAAUCACUUCTT3’.

**1.6 Western blotting**

Total protein was extracted from cells or tissues with RIPA buffer supplemented with protease and phosphatase inhibitor cocktails as previously described.^1^ Protein lysates (20 μg) were separated on SDS‒PAGE and transferred to a polyvinylidene difluoride (PVDF) membrane (#03010040001; Millipore; MA, USA). Membranes were blocked and incubated with primary antibody overnight at 4 °C. Protein bands were visualized with enhanced chemiluminescence (#WBULS0500; Millipore; MA, USA). All primary antibodies applied for western blotting were listed as follows: GAPDH (#60004-1-Ig, 1: 50, 000; Proteintech; IL, USA), PCNA (#13110, 1: 1, 000; Cell Signaling Technology; MA, USA), phospho-PI3K (#17366, 1: 1, 000; Cell Signaling Technology; MA, USA), PI3K (#4249, 1: 1, 000; Cell Signaling Technology; MA, USA), phospho-Akt (#4060, 1: 2, 000; Cell Signaling Technology; MA, USA), Akt (pan) (#4691, 1: 1, 000; Cell Signaling Technology; MA, USA), HTRA1 (#ab274322, 1: 1, 000; Abcam; Cambridge, UK), PCNA (#9532, 1: 1, 000; Cell Signaling Technology; MA, USA), Bcl-2 (#15071, 1: 1, 000; Cell Signaling Technology; MA, USA), Bax (#41162, 1: 1, 000; Cell Signaling Technology; MA, USA), E-cadherin (#20874-1-AP, 1: 20, 000; Proteintech; IL, USA), N-cadherin (#13116, 1: 1, 000; Cell Signaling Technology; MA, USA), Fibronectin (#ab2413, 1: 1, 000; Abcam; Cambridge, UK), ZEB1 (#70512, 1: 1, 000; Cell Signaling Technology; MA, USA), SMAD4 (#46535, 1: 1, 000; Cell Signaling Technology; MA, USA), Vimentin (#5741, 1: 1, 000; Cell Signaling Technology; MA, USA), Cortactin (#ab81208, 1: 50, 000; Abcam; Cambridge, UK), HDAC6 (#7558, 1: 1, 000; Cell Signaling Technology; MA, USA), α-tubulin (#3873, 1: 1, 000; Cell Signaling Technology; MA, USA), acetyl-α-tubulin (#5335, 1: 1, 000; Cell Signaling Technology; MA, USA).

**1.7 Immunoprecipitation**

Immunoprecipitations were performed with the Pierce^TM^ Classic Magnetic Immunoprecipitation (IP)/Co-IP Kit (#88804; ThermoFisher Scientific; MA, USA) following the manufacturer’s instructions. The primary antibody against HTRA1 (#ab274322; Abcam; Cambridge, UK) was used at a concentration of 1:30. Immunoprecipitated proteins were analyzed on western blots.

**1.8 3D tumor spheroid invasion assay**

The 3D tumor spheroid invasion assay was performed with a kit (#3500-096-k; Trevigen; MD, USA) following the manufacturer’s instructions. Images of the tumor spheroids were obtained every 24 h using an inverted microscope (Nikon; Tokyo, Japan) and analyzed with the software ImageJ (NIH, MD, USA).

**1.9 Transwell assay**

The properties of migration and invasion were examined in transwell assays performed as previously described.^3^ The cells were transfected with siRNA, and after 48 h, 2 × 10^4^ cells were seeded into the transwell chambers (#3422; Corning; NY, USA). Chambers were incubated for 48 h, and the migrated or invaded cells were fixed with formalin and stained with hexamethylpararosaniline (#C0121; Beyotime Biotechnology; Shanghai, China). Cells were counted in four random fields obtained from each chamber with an Olympus inverted microscope.

**1.10 Wound healing assay**

LN229 or U251MG cells were inoculated into 6-well plates. When cell confluence reached approximately 95%, cells were transfected with siRNAs. A wound was immediately created by scratching the monolayer with a 200 μL pipette tip. Images of cells were acquired at 0, 24 and 48 h after wounding the monolayers.

**1.11 Cell proliferation assays**

Cell Counting Kit-8 (CCK-8) (#CA1210, Solarbio; Beijing, China) and EdU assays (#C10310-1; RiboBio; Guangzhou, China) were performed following the manufacturers’ instructions to assess cell proliferation. EdU-positive cells were acquired with an inverted microscope (Olympus; Tokyo, Japan) and counted with the software ImageJ (NIH, Bethesda, MD, USA).

**1.12 F-actin labeling**

Cells were transfected with HTRA1 siRNAs, and after 48 h, fixed in formalin. The F-actin cytoskeleton was stained with ActinRed™ 555 ReadyProbes (#R37112; ThermoFisher Scientific; MA, USA), and the cell nucleus was stained with DAPI (#P0131; Beyotime Biotechnology; Shanghai, China) as previously described.^1^ Images were obtained with an inverted microscope (Nikon; Tokyo, Japan).

**1.13 Immunofluorescence staining**

LN229 or U251MG cells were inoculated into 8-well confocal plates. After transfection with siRNA or overexpression plasmids, cells were fixed with 4% paraformaldehyde and permeabilized with 0.4% Triton X-100 in PBS. Then cells were blocked with 5% bovine serum albumin (A8020, Solarbio; Beijing, China) and incubated with primary antibody against HDAC6 (#7558, 1: 200; Cell Signaling Technology; MA, USA) as well as α-tubulin (#3873, 1: 1, 000; Cell Signaling Technology; MA, USA) overnight at 4 ℃. Then the primary antibody was visualized with two secondary antibodies: Alexa Fluor 488-conjugated goat anti-rabbit IgG (#ab150077, 1: 200; Abcam; Cambridge, UK) and Alexa Fluor 594-conjugated goat anti-mouse IgG (#ab150116, 1: 200; Abcam; Cambridge, UK). The nucleus was stained with DAPI (#P0131; Beyotime Biotechnology; Shanghai, China). Images were obtained with confocal microscopy system (Leica; Wetzlar, Germany).

**1.14 Flow cytometry**

Cells transfected with siRNA were collected by centrifugation. For apoptosis analysis, cells were stained with propidium iodide (PI) and Annexin V-FITC (#556547; BD Biosciences; CA, USA) to label apoptotic cells according to the manufacturer’s instructions, and detected with flow cytometry on a BD Accuri C6 flow cytometer (BD Biosciences; San Diego, CA, USA). For the analysis of cell cycle, cells were fixed in 75% ethanol for 24 hours. Then fixed cells were stained with PI/RNase staining buffer (#550825; BD Biosciences; CA, USA) according to manufacturer’s protocol and detected with the BD Accuri C6 flow cytometer (BD Biosciences; CA, USA). The results were analyzed with MODFIT LT 4.0 (Verity Software House Inc.; Topsham, ME, USA).

**1.15 Transcriptome sequencing**

GBM#P3 cells transfected with Lenti-Control or Lenti-shHTRA1 were harvested and immediately frozen in liquid nitrogen for subsequent sequencing. Total RNA was isolated with TRIzol. Briefly, the cells were harvested by centrifugation at 200 g, 4 ℃ for 5 minutes. Then 1ml TRIzol was added to cells and pipetted 20 times to make sure all cells were lysed. Transcriptome sequencing was performed on 1 µg of total RNA at Lc-Bio Technologies Co., Ltd. (Hangzhou, China). Genes with |log_2_FoldChange| > 1 and FDR < 0.05 were regarded as differentially expressed genes.

**SUPPLEMENTARY FIGURE LEGENDS**

**Figure S1. A risk score model based on serine proteases’ expression predicts the prognosis of glioma patients.** (A-B) The risk score distribution in train (A, n = 347) and test (B, n = 347) data. The train and test data are randomly selected from all glioma data and each group has 50% of all data. (C-D) The distribution of dead and alive samples is displayed with risk score in train (C) and test (D) data. (E-F) The survival curves of high and low risk score samples train (E) and test (F) data. (G-H) The ROC curves predicting the survival of glioma patients based on risk score model in train (G) and test (H) data. (F) and (G) log-rank test. ***, *P* < 0.001.

**Figure S2. A risk score model based on serine proteases’ expression predicts the prognosis of GBM patients.** (A-B) The survival curves of high and low risk score samples train (A, n = 84) and test (B, n = 83) data. The train and test data are randomly selected from all GBM data and each group has 50% of all data. (C-D) The ROC curves predicting the survival of GBM patients based on risk score model in train (C) and test (D) data. (A) and (B) log-rank test. **, *P* < 0.01.

**Figure S3. The expression levels of all 52 PRSS family genes and their correlations with prognosis.** (A) The vioplot showing expression levels of all 52 PRSS family genes. PRSS10 (TMPRSS2) and PRSS7 (TMPRSS15) were included because PRSS10 was reported to be a soluble protease and PRSS7 was confirmed to participate in microvilli formation, according to Uniprot database. Normal brain, n = 5; glioma, n = 698. (B) The heatmap showing the correlation between serine protease expression and clinical features. n = 698. (C) Serine protease genes that are significantly associated with survival of glioma patients. (A), Student’s t test. *, *P* < 0.05; **, *P* < 0.01; ***, *P* < 0.001.

**Figure S4. The correlation between HTRA1 level and immune infiltration.** (A) The vioplot of infiltrating immunocytes’ ratios in TCGA glioma samples (n = 694) calculated with CIBERSORT algorithm. (B) The correlations between HTRA1 expression level and ESTIMATEScore, ImmuneScore, StromalScore and Tumor Purity, respectively. These four indexes are calculated with “ESTIMATE” package in R. (C) The ESTIMATEScore, ImmuneScore, StromalScore and Tumor Purity in high and low HTRA1 groups. (A) and (C), Student’s t test; (B), Pearson’s correlation analysis. *, *P* < 0.05; **, *P* < 0.01; ***, *P* < 0.001.

**Figure S5. Knockdown of HTRA1 caused G0/G1 cell cycle arrest.** (A) Representative images showing the cell cycle distribution after treating with siNC or siHTRA1 siRNAs. The images were obtained from MODFIT LT 4.0 by analyzing flow cytometry results. (B) Statistical analysis of cell proportion of G0/G1 and G2/M cell cycle. (B), one-way ANOVA, post hoc Dunnett’s test. *, *P* < 0.05; **, *P* < 0.01; ***, *P* < 0.001.

1. Wang H, Chen Z, Wang S, et al. TGFβ1-induced beta-site APP-cleaving enzyme 2 upregulation promotes tumorigenesis through the NF-κB signalling pathway in human gliomas. *Molecular oncology.* 2020;14(2):407-425.

2. Muñoz SS, Li H, Ruberu K, et al. The serine protease HtrA1 contributes to the formation of an extracellular 25-kDa apolipoprotein E fragment that stimulates neuritogenesis. *The Journal of biological chemistry.* 2018;293(11):4071-4084.

3. Qian M, Wang S, Guo X, et al. Hypoxic glioma-derived exosomes deliver microRNA-1246 to induce M2 macrophage polarization by targeting TERF2IP via the STAT3 and NF-κB pathways. *Oncogene.* 2020;39(2):428-442.
